# Exploratory insights into prefrontal cortex activity in continuous glucose monitoring: findings from a portable wearable functional near-infrared spectroscopy system

**DOI:** 10.3389/fnins.2024.1342744

**Published:** 2024-05-08

**Authors:** Jiafa Chen, Kaiwei Yu, Songlin Zhuang, Dawei Zhang

**Affiliations:** Research Center of Optical Instrument and System, Ministry of Education and Shanghai Key Lab of Modern Optical System, University of Shanghai for Science and Technology, Shanghai, China

**Keywords:** the prefrontal cortex, functional near-infrared spectroscopy, blood glucose levels, continuous monitoring, predictive modeling

## Abstract

The escalating global prevalence of diabetes highlights an urgent need for advancements in continuous glucose monitoring (CGM) technologies that are non-invasive, accurate, and user-friendly. Here, we introduce a groundbreaking portable wearable functional near-infrared spectroscopy (fNIRS) system designed to monitor glucose levels by assessing prefrontal cortex (PFC) activity. Our study delineates the development and application of this novel fNIRS system, emphasizing its potential to revolutionize diabetes management by providing a non-invasive, real-time monitoring solution. Fifteen healthy university students participated in a controlled study, where we monitored their PFC activity and blood glucose levels under fasting and glucose-loaded conditions. Our findings reveal a significant correlation between PFC activity, as measured by our fNIRS system, and blood glucose levels, suggesting the feasibility of fNIRS technology for CGM. The portable nature of our system overcomes the mobility limitations of traditional setups, enabling continuous, real-time monitoring in everyday settings. We identified 10 critical features related to blood glucose levels from extensive fNIRS data and successfully correlated PFC function with blood glucose levels by constructing predictive models. Results show a positive association between fNIRS data and blood glucose levels, with the PFC exhibiting a clear response to blood glucose. Furthermore, the improved regressive rule principal component analysis (PCA) method outperforms traditional PCA in model prediction. We propose a model validation approach based on leave-one-out cross-validation, demonstrating the unique advantages of K-nearest neighbor (KNN) models. Comparative analysis with existing CGM methods reveals that our paper’s KNN model exhibits lower RMSE and MARD at 0.11 and 8.96%, respectively, and the fNIRS data were highly significant positive correlation with actual blood glucose levels (*r* = 0.995, *p* < 0.000). This study provides valuable insights into the relationship between metabolic states and brain activity, laying the foundation for innovative CGM solutions. Our portable wearable fNIRS system represents a significant advancement in effective diabetes management, offering a promising alternative to current technologies and paving the way for future advancements in health monitoring and personalized medicine.

## 1 Introduction

The escalating prevalence of diabetes globally accentuates the critical need for effective management strategies, central to which is the monitoring of blood glucose levels (Phillip et al., 2023). Continuous glucose monitoring (CGM) systems have revolutionized diabetes care, offering real-time insights into glucose dynamics, facilitating tighter glucose control, and reducing the risk of complications (Lee et al., 2021). Despite significant advancements, the quest for non-invasive, accurate, and patient-friendly CGM technologies remains at the forefront of diabetes research.

Presently, prevailing methodologies for glucose monitoring encompass approaches like blood sampling through puncture, subcutaneous implantable glucose monitors, cumulative assays, and optical continuous tracking. Regrettably, these approaches have limitations—ranging from invasiveness and discomfort to inadequate sampling frequency, inability to sustain continuous monitoring, and prohibitive costs (Butler et al., 2023). In this context, functional near-infrared spectroscopy (fNIRS) emerges as a promising tool 4. fNIRS, a non-invasive optical imaging technique, measures hemodynamic responses associated with neuronal activity, providing insights into the underlying mechanisms of various physiological and pathological conditions (Rebelos et al., 2023). Its application in cognitive neuroscience has been particularly noteworthy, enabling researchers to explore the neural correlates of a wide range of cognitive functions (Pinti et al., 2020). The potential of fNIRS to investigate the neural basis of glucose metabolism and its regulation presents an exciting avenue for research, especially in the context of diabetes and metabolic disorders.

The prefrontal cortex (PFC) plays a pivotal role in cognitive functions, including decision-making, impulse control, and the regulation of emotional responses (Ngetich et al., 2020). Functionally connected to the hypothalamus and brainstem, the PFC intricately participates in the human system’s endocrine, metabolic, and appetite regulation, thereby assuming a critical mantle in maintaining stable blood glucose levels (de Lima et al., 2022). Furthermore, the PFC extends its influence on blood glucose levels by engaging in intricate neural networks with other cerebral regions. This dynamic interplay significantly shapes higher-order mental activities like cognition, emotion, and behavior (Micha et al., 2011; Lucas et al., 2019). Previous studies have highlighted the PFC’s role in inhibiting food intake and its sensitivity to changes in glucose levels, suggesting a complex interplay between cognitive functions and metabolic states (Tataranni et al., 1999; Page et al., 2011). In addition, related studies have demonstrated the PFC’s response to hypoglycemia and its role in the counterregulatory response to low blood glucose levels (Choudhary et al., 2018; Nwokolo et al., 2019), underscoring the significance of understanding PFC activity in the context of glucose monitoring. The rationale for focusing on the PFC stems from its critical role in executive functions directly relevant to dietary behavior and glucose regulation (Dohle et al., 2018). The PFC’s ability to integrate various types of sensory information, including metabolic signals, and its involvement in complex decision-making processes makes it an ideal candidate for studying the neural basis of CGM. Furthermore, during fasting, blood glucose levels naturally decline, a shift influenced by dietary intake and the intricate mechanisms governing blood glucose regulation within the body (Kumar et al., 2023). Hypoglycemia, characterized by inattention, dizziness, rapid heart rate, cognitive impairment, and mood fluctuations, profoundly impacts the PFC’s functionality (McCrimmon, 2021; Zaremba et al., 2023). In the transition to the glucose state, the relevant characteristics are improved, which further affects PFC function. This physiological modulation is intricately tied to the adjustments within the optical properties of the PFC, an interplay that underscores the dynamic influence of blood glucose levels on cerebral dynamics. The complex interplay between hemoglobin levels and blood glucose concentrations within the PFC is a multifaceted orchestration involving an array of interconnected mechanisms, all subject to regulation by various influential factors. Notably, alterations in blood glucose levels directly impact the functional dynamics of the PFC, thereby rippling across domains like cognition, emotion, and decision-making, encapsulating diverse dimensions of brain activity (Deco et al., 2023). Reciprocally, the activity and function of the PFC loop back to exert a feedback influence on blood glucose regulation and HbO. This intricate and bidirectional dance underscores how fluctuations in blood glucose levels directly affect hemoglobin concentrations within the PFC, subsequently imprinting their mark on the response of fNIR light regarding hemoglobin’s absorption and scattering properties. It is imperative to highlight that the PFC’s role in blood glucose regulation is a realm of enduring interest within neuroscience.

It is noteworthy that the current fNIRS system gathers data via wired connections. The cumbersome nature of this connection method and the stationary desktop setup impede the system’s flexibility, constraining the practical application of fNIRS technology (Torricelli et al., 2014). Moreover, optimizing several critical aspects in the design of fNIRS systems is imperative: firstly, refining the layout design of electrodes and optodes is necessary; secondly, embracing a lightweight, highly integrated design is essential to enhance user experience; ensuring the acquisition of high-quality signals is pivotal; adopting an ergonomic design is crucial to provide comfortable wearing experience; developing portable, wearable, lightweight, and flexible devices is imperative; cost reduction, enhancing equipment complexity and reliability, as well as advancing resolution and functionality, are also vital considerations. Hence, developing a novel integrated portable fNIRS system for monitoring blood glucose is essential.

This study utilizes a novel wearable portable fNIRS imaging system to explore the activity of the PFC in CGM. Our lightweight modular design addresses the mobility limitations of previously fixed setups, with critical technical parameters outlined in Table 1. By integrating fNIRS technology with CGM data, we aim to elucidate the role of the PFC in glucose regulation and evaluate fNIRS as a potential tool for monitoring the link between metabolic status and neural activity. However, despite the enormous potential of utilizing fNIRS to explore PFC activity in CGM, our research is currently still in its exploratory and conceptual validation stage. This means that further studies are needed to confirm its feasibility and accuracy, as well as to validate the reproducibility and robustness of the results obtained. This stage requires continuous experimental design and data validation to ensure that the methods and techniques employed can be robustly applied in various real-world settings and study populations. Additionally, a deeper understanding of the relationship between PFC activity and blood glucose dynamics is required, along with exploring its potential role in diabetes management and treatment. Therefore, for research in this field, we still need to conduct more experiments and clinical studies to comprehensively assess its prospects and potential benefits in clinical practice.

**Table 1:** fNIRS system main technical specifications.

Technical indicators	Parameters
Measurement projects	21 channels
Spectral type	Successive waves
Wavelength	760 nm, 830 nm
Source-probe quantity	10, 8 (weight ≤12 g)
Data transmission	Bluetooth, in real-time transmission distance of 30 m
Sampling frequency	≤150 Hz
fNIRS host size and weight	8.5 × 8.5 × 3.5 cm, ≤300 g
Light source type	LED
Detector type	SiPDs; sensitivity (<1 pW); dynamic range (≥90 dB)
fNIRS battery part	1. Power adapter: input is 100 V-240 V, 50/60 Hz, output is 5 V 2. Battery type: it has a built-in lithium battery that can be supplemented with external batteries and power banks to extend battery life 3. Battery dimensions: 3 × 2.5 × 0.6 cm 4. Battery capacity: 1400 mAH 5. Battery output voltage: 3.7 V 6. Battery efficiency: 93% 7. Battery endurance time: ≥6 h
Functions	One-stop data preprocessing, event and data editing, artifact removal/correction, probe position editing, dynamic display of oximetry status, GLM, fast real-time display of 2D mapping maps, support for display of HbO, HbR, Hb status, signal quality detection in 2D, scalp, cerebral cortex, and glass view, etc.

## 2 Materials and methods

### 2.1 Participants

In this study, we enrolled 15 healthy university students (eight males and seven females, ranging in age from 19 to 28 years, with a mean age of 23.35 ± 2.3 and a mean body mass index of 23.17 kg/m^2^). All participants were affiliated with University of Shanghai for Science and Technology and demonstrated a strong willingness to collaborate. We excluded individuals with acute physical conditions, neurological disorders, history of drug abuse, or chronic or acute diseases that impair brain function, including but not limited to diabetes, renal failure, arrhythmias, and other cardiovascular diseases. The study protocol was approved by the Medical Ethics Committee of Peking University’s Sixth Hospital and University of Shanghai for Science and Technology Affiliated Shidong Hospital (approval number 2022371), adhering to the Declaration of Helsinki’s guidelines (Stockhausen, 2000) as stipulated by the World Medical Association. Before the experiment, all participants provided written informed consent and were fully briefed on their right to discontinue participation at any point.

### 2.2 Experimental design

To initiate, before the experimental proceedings, meticulous health assessments and psychological orientation were administered to the participants by certified medical professionals, safeguarding their physiological and psychological suitability for active engagement in the study. Before the start of the experiment, participants wore a near-infrared brain function meter and were observed for 5 min to ensure that the blood flow signals in the fNIRS channel were at a steady state. Throughout the experimental protocol, participants sat in a comfortable chair. They were instructed to relax, not to move, speak, or blink too much to avoid noise and stabilize the blood flow in fNIRS channels. The experimental schema was bifurcated into two discernible phases: fasting and the glucose states.

All participants underwent a fasting regimen during the fasting phase, observing a minimum fasting duration of 5 h before the experiment’s initiation. Subsequently, the participants’ PFC was subjected to continuous monitoring via the fNIRS imager, spanning 10 min. Simultaneously, participants underwent a fingertip blood collection operation, culminating in the acquisition of congruent glucose measurements to serve as a control cohort for validation.

The second stage was the glucose state. All participants drank 50 mL of glucose solution based on their fasting state. Then, the fNIRS imager continuously monitored the participants’ PFC for 60 min to obtain their fNIRS data in the glucose state. Participants underwent fingertip blood collection as a control group to get the corresponding blood glucose values.

In this study, we implemented a unique experimental design where each participant served in dual roles as both the experimental subject and their control. The control condition involved monitoring blood glucose levels through fingertip blood sampling, facilitating a direct comparison between the precision and effectiveness of fNIRS technology and conventional blood glucose monitoring techniques while accounting for individual differences. We obtained fNIRS data and blood glucose levels in fasting and glucose-drinking states using fNIRS imager and a blood collection device, further validating the feasibility of using fNIRS to monitor blood glucose levels in PFC.

### 2.3 Overview of the new portable wearable fNIRS system

**Figure 1:**
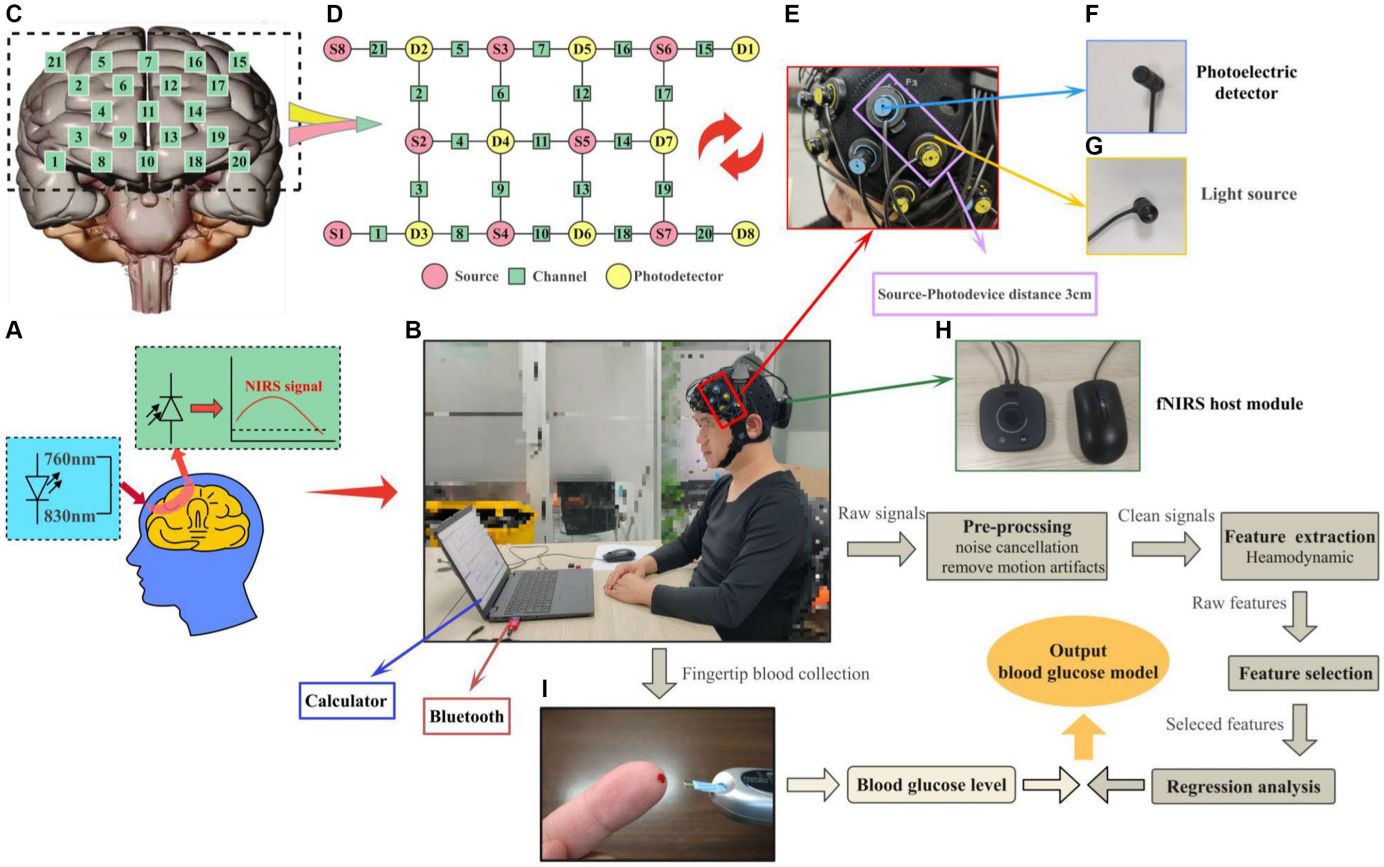
Schematic diagram of the portable wearable fNIRS system and visualization of the experimental process. **(A)** fNIRS experimental principle. **(B)** Flowchart of the experimental setup and data processing. **(C)** Layout of the number of channels in the PFC. **(D)** Layout of the number of channels in the source-probe-channel. **(E)** Source-probe device. **(F)** Photoelectric detector. **(G)** Light source. **(H)** fNIRS host module. **(I)** Fingertip glucose test.

Our independently designed system comprises custom-made full-head caps, light sources, photodetectors, integrated miniaturized fNIRS host modules, and components. The entire equipment weighs ≤300 g, with a built-in rechargeable lithium battery, adopting a lightweight, integrated design that places the whole system on the head without needing a backpack. This achieves true portability, is suitable for indoor and outdoor experiments, and ensures participant comfort during activities, as shown in Figure 1. Additionally, the fNIRS experimental setup used in this study comprises 10 emitting light sources (wavelengths 760 nm and 830 nm), eight detectors, and an integrated miniaturized fNIRS host module (dimensions: 8.5 × 8.5 × 3.5 cm, weight ≤300 g), totaling 21 channels, as illustrated in Figures 1B,D,E,H. The system’s light source section adopts an LED ring pin design (weight <12 g) and integrates a custom ring buckle fixator to shield ambient light, as depicted in Figure 1G. The light source employs transistor-driven circuits, with control using dual-wavelength LEDs employing time-division multiplexing technology. The system’s photodetectors utilize a photodiode ring pin design (weight <12 g) and feature a custom ring buckle bracket for convenient light reception, as shown in Figure 1F. These detectors integrate avalanche photodiode operational amplifiers on the same chip, enabling direct conversion of light signals into voltage signal outputs, achieving high-gain, low-noise amplification of weak signals. Moreover, we employ synchronous modulation technology between LED light sources, photodetectors, and digital smoothing algorithms to stabilize detector operation under solid ambient light interference. We integrate light source drive circuits, amplification and filtering circuits, I/V conversion circuits, A/D circuits, wireless communication circuits, ARM processing circuits, and power circuits in the fNIRS host module using low-temperature co-fired ceramic technology, encapsulated in a 3D-printed plastic shell. This process adheres to the principles of a minor, portable, comprehensive system featuring stable and efficiently designed circuits. It controls units such as near-infrared spectrum signal acquisition, light source drive, power supply, and data transmission, as shown in Figures 1B,H. The system employs a flexible fiber cap as a foundation, with perforations fixing the fNIRS light source-detector brackets in specific positions. The spatial range is adjusted via elastic joints to maintain a distance of 30 mm between the light source and detector, carefully positioned uniformly over the PFC (10–20 system), enveloping the PFC surface for near-infrared spectrum data collection, as illustrated in Figure 1C.

The upper computer processing platform plays a crucial role in the system, encompassing tasks such as data communication, data processing, display interface design, and brain function recognition analysis. This section is integrated using Python 3.8 and Visual Studio 2022, with Visual Studio handling tasks such as data communication, data processing, and display interface design. Python handles data calculation, feature extraction, and classification recognition training tasks. The serial port connects the data communication segment to the hardware detection platform. The communication content mainly includes three parts: configuring the communication port, verifying the functionality of the communication port by sending different signals to control the startup, pause, and stop of the hardware platform, and receiving and parsing fNIRS data packets. The hardware platform sends data to the upper computer, where the data is parsed and verified, separating near-infrared data channels based on the timing of light signals and data collection. During the data parsing, weak signals of different wavelengths are divided based on timing. Subsequent data processing involves a systematic sequence, including preprocessing, feature analysis and selection, feature extraction, and finally, the construction of prediction models, as depicted in Figure 1B. Additionally, fingertip blood glucose values were obtained from the control group, as shown in Figure 1I.

#### 2.3.1 Data preprocessing

In the fNIRS system, the most fundamental electrical signal is the change in optical intensity measured by photodetectors in brain tissue, reflecting the absorption and scattering of near-infrared light by brain tissue, which can be used to reveal changes in cerebral blood flow and the oxygenation status of hemoglobin (Liu et al., 2022). Preprocessing this data is a crucial step in near-infrared calibration, aimed at suppressing various noise and artifacts, enhancing the signal-to-noise ratio, and more accurately estimating the hemodynamic response induced by functional activities (Patashov et al., 2023). This study employs wavelet transformation for data processing, which features a time-frequency window, allowing for more precise analysis of local information, especially effective in handling sharp peak artifact noise and drift trends (Duan et al., 2018). Through multiscale decomposition, wavelet transformation provides more comprehensive information in both time and frequency domains, eliminating motion artifacts (Perpetuini et al., 2021).

**Figure 2:**
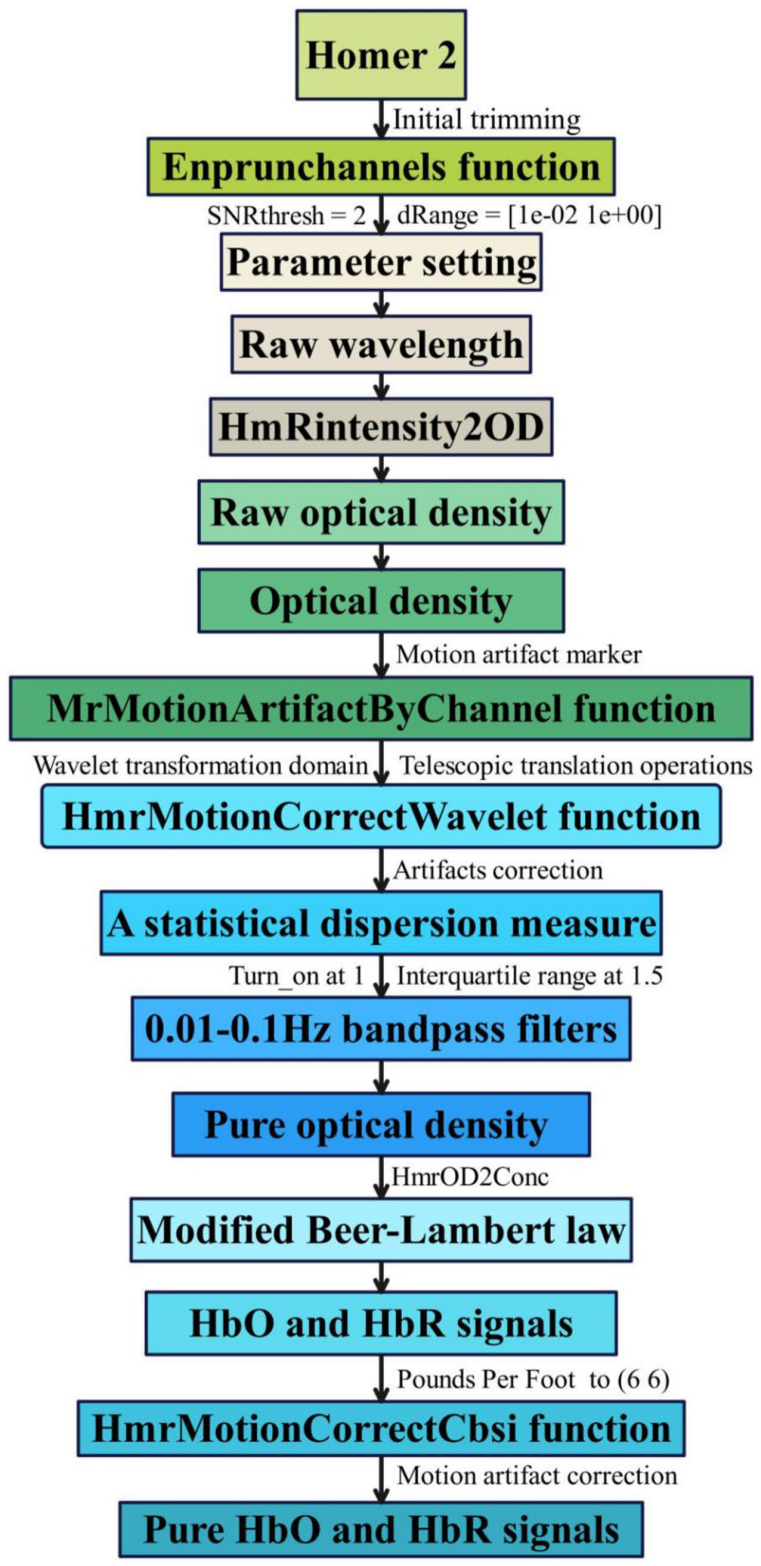
fNIRS data preprocessing flowchart.

**Figure 3:**
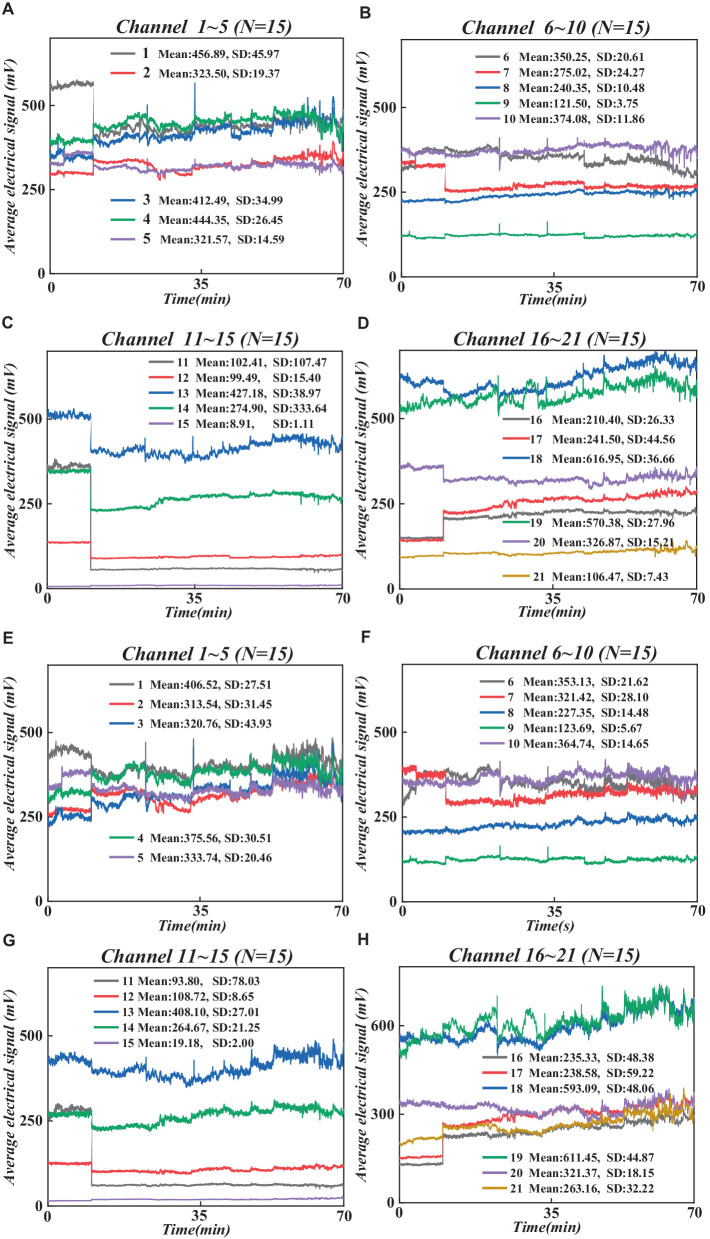
Raw wavelength averages for all participants under 21 channels. **(A)** 760 nm raw wavelength averages under channel 1–5. **(B)** 760 nm raw wavelength averages under channel 6–10. **(C)** 760 nm raw wavelength averages under channel 11–15. **(D)** 760 nm raw wavelength averages under channel 16–21. **(E)** 830 nm raw wavelength averages under channel 1–5. **(F)** 830 nm data under channel 6–10. **(G)** 830 nm wavelength data under channel 11–15. **(H)** 830 nm data under channel 16–21. **(F)** 830 nm raw wavelength averaged over 6–10 channels. **(G)** 830 nm raw wavelength averaged over 11–15 channels. **(H)** 830 nm raw wavelength averaged over 16–21 channels.

We utilized the MATLAB-based graphical user interface program Homer 2 (Huppert et al., 2009) toolkit for analyzing and processing fNIRS data, as depicted in Figure 2. We conducted an automated data quality check (initial trimming) using the enprunchannels function. During this process, channels with excessively weak or solid signals or large standard deviations were removed from the measurement list to ensure data quality. Subsequently, we defined the data extraction parameters [dRange: (1 × 10^−2^ to 1 × 10^0^), signal-to-noise ratio: SNRthresh = 2], obtaining average data for the raw wavelengths of all participants under 21 channels is shown in Figure 3.

**Figure 4:**
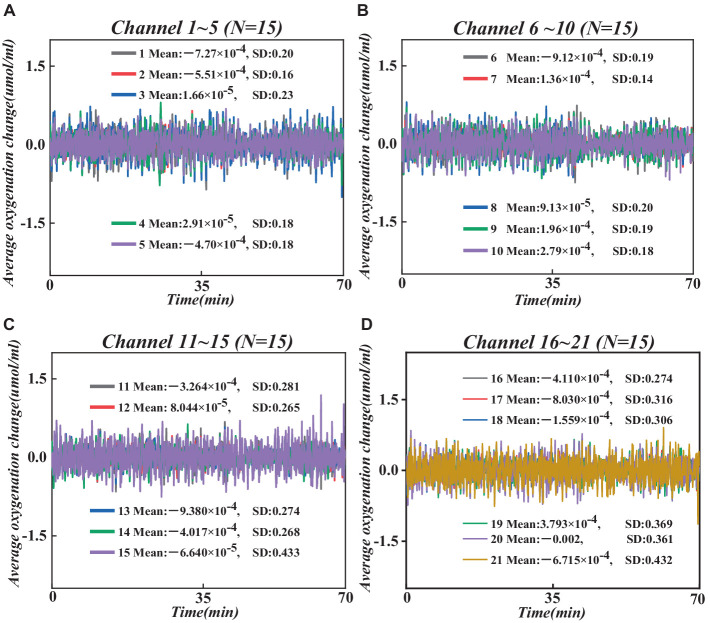
Changes in average HbO concentration across all participants at 21 channels. **(A)** Changes in average HbO concentration in channels 1–5. **(B)** Changes in average HbO concentration in channels 6–10. **(C)** Changes in average HbO concentration in channels 11–15. **(D)** Changes in average HbO concentration in channels 16–21.

Next, we converted the raw wavelength average data into raw optical density (OD) average data using the hmRintensity2OD function, which characterizes light intensity in brain tissue, reflecting changes in cerebral oxygenation levels and blood flow (Wang et al., 2018). Then, the mrMotionArtifactByChannel function was employed to detect and label artifacts on all channels systematically. Based on these labeled artifacts, we performed motion artifact correction using the hmrMotionCorrectWavelet function and statistical dispersion based on the wavelet transform domain, followed by the application of a bandpass filter (0.01–0.1 Hz) to remove random noise induced by instrumentation and physiological noise caused by heart rate/respiration (Bizzego et al., 2020), thereby obtaining pure OD average data. Subsequently, we employed the modified Beer–Lambert law (Nikzad-Langerodi et al., 2020) to convert the filtered pure OD average data into changes in the average concentrations of hemoglobin oxygenation (HbO) and deoxyhemoglobin (HbR), reflecting cerebral oxygenation levels and serving as indicators of PFC functional activity. To further enhance data quality, we utilized the hmrMotionCorrectCbsi function. We employed a correlation-based signal enhancement method to process changes in HbO and HbR concentrations for further motion artifact correction, resulting in pure HbO and HbR signals (Chen et al., 2024). Also, based on relevant studies, HbO is more sensitive to brain activity, and its signal sensitivity and reliability are higher than HbR (Swethasri et al., 2017). Therefore, this study analyzes the average HbO signal of all participants (see Figure 4).

To screen the channels of interest more accurately from the 21 channels, we performed *t*-tests on the change in mean HbO concentration in two independent samples from all participants in fasting and glucose states, and the results are shown in Table 2.

**Table 2:** *t*-test and variance results for 21 channels for all participants based on predefined conditions.

Channel	Fasting state variance	Glucose states variance	*t*	*p*
1	0.208	0.203	−1.01	0.156
2	0.154	0.160	−0.92	0.18
3	0.221	0.231	−0.406	0.343
4	0.176	0.184	0.0837	0.467
5	0.170	0.177	0.899	0.184
6	0.190	0.186	0.666	0.253
7	0.138	0.141	−1.91	0.0278
8	0.217	0.195	0.258	0.395
9	0.195	0.186	−0.179	0.429
10	0.204	0.173	−0.0500	0.480
11	0.170	0.171	−0.73	0.233
12	0.151	0.148	−2.45	0.00713
13	0.156	0.148	0.973	0.165
14	0.170	0.152	−1.28	0.0997
15	0.224	0.227	−2.3	0.0107
16	0.168	0.147	−1.13	0.127
17	0.181	0.165	−1.28	0.101
18	0.179	0.170	1.66	0.0483
19	0.196	0.195	−1.77	0.0382
20	0.247	0.220	1.03	0.151
21	0.213	0.233	0.179	0.429

Observing Table 2, it becomes evident that instances where *p* < 0.05 indicates a noteworthy discrepancy in the alteration of mean HbO concentration between the fasting and glucose states within the specific channel. Among the 21 initially considered channels, 7 possessed significant differentials. These channels, specifically the 7th, 12th, 15th, 18th, and 19th, stood out as contributors to notable variations in mean HbO concentration dynamics. By harnessing the insights garnered from these significant channels, it is anticipated that a refined data extraction process minimizes experimental error while optimizing experimental efficiency.

### 2.4 Feature extraction and selection

#### 2.4.1 Feature analysis

Following completion of the data processing, this study extracted 10 critical characteristic features of mean HbO concentration variations from seven notable channels in the fasting and glucose states, which include mean (*λ*_1_), standard deviation (*λ*_2_), mean (*λ*_3_), and standard deviation (*λ*_4_) of the first-order difference, mean (*λ*_5_) and standard deviation (*λ*_6_) of the second-order difference, peak (*λ*_7_), peak-to-peak (*λ*_8_), energy (*λ*_9_), and energy entropy (*λ*_10_).

*λ*_1_ represents the mean of the change in mean HbO concentration over the monitoring time, and this feature reflects the average activity level profile of the PFC in the fasting and glucose states.


$$\lambda_{1}=\frac{1}{T}\overset{T}{\underset{i=1}{\sum }}x_{i}$$

where T is the number of time points, and $x_{i}$ represents the change in mean HbO concentration within the first time point. A measure of the degree of change in mean HbO concentration can be obtained as the standard deviation *λ*_2_. This feature reflects the fluctuations in mean HbO concentration in the PFC in the fasting and glucose states.


$$\lambda_{2}=\sqrt{\frac{\sum^{\underset{T}{i=1}}{\left(x_{i}-\lambda_{1}\right)}^{2}}{T}}$$

*λ*_3_ is the mean of the first-order difference, which represents the average rate of change of mean HbO concentration in the fasting and glucose states and is used to characterize the dynamics of activity in the PFC.


$$\lambda_{3}=\frac{1}{T-1}\overset{\underset{T}{i=2}}{\sum }\left(x_{i}-x_{i-1}\right)=\frac{1}{T-1}\overset{\underset{T}{i=2}}{\sum }\Delta x$$

where Δx is the difference in mean HbO concentration between adjacent periods. The standard deviation *λ*_4_ of the first-order difference series can be obtained, which reflects the dynamic range of mean HbO concentrations in the PFC in the fasting and glucose states.


$$\lambda_{5}=\frac{1}{T-2}\overset{\underset{T}{i=3}}{\sum }\left(x_{i}-2x_{i-1}+x_{i-2}\right)$$

We obtain *λ*_6_, the standard deviation of the second-order difference, a feature that reflects the degree of fluctuation in the rate of change of mean HbO concentration in the PFC in the fasting and glucose states.


$$\lambda_{6}=\sqrt{\frac{1}{T-3}\overset{T}{\underset{i=3}{\sum }}\left(x_{i}-2x_{i-1}+x_{i-2}-\lambda_{5}\right)}$$

*λ*_7_ is the maximum value of the mean HbO concentration change, and this feature reflects the strongest response and the significant response of the PFC activity in the fasting and glucose states.


$$\lambda_{7}=\psi_{\text{max}}$$

where $\psi_{\text{max}}$ is the maximum value of mean HbO concentration during the monitoring time. The difference between the peak and the minimum value of the HbO concentration, i.e., the peak-to-peak value *λ*_8_ is obtained, and this feature reflects the maximum fluctuation amplitude of the mean HbO concentration of the PFC in the fasting and glucose states.


$$\lambda_{8}=\psi \min_{\text{max}}$$

where $\psi_{\text{min}}$ is the minimum value of mean HbO concentration during the monitoring time. *λ*_9_ represents the total energy of the mean HbO concentration, a feature used to characterize the intensity of the PFC activity in the fasting and glucose states.


$$\lambda_{9}=\overset{0}{\underset{t}{\int }}{\left(\Delta_{HbO}\right)}^{2}dt$$

where: $\Delta_{HbO}$ denotes the change in concentration over the period; t denotes the length of the period. The energy entropy *λ*_10_ represents a measure of uncertainty in the energy distribution of the signal. It is used to characterize the complexity and stochasticity of the mean HbO concentration in the PFC in the fasting and glucose states.


$$\lambda_{10}=-\overset{0}{\underset{t}{\int }}{\left(\Delta_{HbO}\right)}^{2}*\ln\left[{\left(\Delta_{HbO}\right)}^{2}\right]dt$$

#### 2.4.2 Feature extraction and selection

The primary objective of this study is to employ principal component analysis (PCA) techniques for feature extraction from mean HbO levels in both fasting and glucose states of fNIRS data, resulting in a novel set of feature sets exhibiting significant outcomes (Fujiwara et al., 2022). This approach offers a distinct advantage by significantly reducing the data’s complexity, thereby enhancing the efficiency of subsequent analyses. However, it is essential to note that there is a potential trade-off, as some critical feature information may need to be recovered during the dimensionality reduction process. Thus, in this study, our approach extends beyond applying the traditional PCA algorithm; we conduct in-depth refinements and optimizations to ensure that the new feature vectors possess more comprehensive and accurate information expression capabilities.

We have succeeded in effectively dividing the corresponding dataset into training and testing sets based on the corresponding dataset, and further set up a training matrix Γ , based on the training set, which can be obtained:


$$\Gamma \in {Η}^{a\times b}$$

where a is the number of training sets and b is the dimension of the training sets. Based on this setting, we can derive the average value of the number of training sets:


$${\bar{x}}_{tra}=\frac{1}{a}\overset{a}{\underset{j=1}{\sum }}x_{j}$$

where $x_{j}$ is the j th training set. We can get the covariance matrix of this training set:


$$\Pi =\overset{a}{\underset{k=1}{\sum }}\left(x_{k}-{\bar{x}}_{tra}\right){\left(x_{k}-{\bar{x}}_{tra}\right)}^{\Phi }$$

where $x_{k}$ is the number of the k th training set and Φ is the matrix device. it can be obtained by effective feature decomposition of the training matrix:


$$\Pi \times C_{a}=\lambda_{a}\times C_{a}$$

where $C_{1}$ , $C_{2}$ , $C_{3}$ … $C_{a}$ are a set of eigenvectors, and $\lambda_{1}$ , $\lambda_{2}$ , $\lambda_{3}$ … $\lambda_{a}$ are the corresponding eigenvectors. According to the result of eigen decomposition, we can obtain the intraclass scattering matrix:


$$\Pi_{ICS}=\overset{n}{\underset{i=1}{\sum }}\overset{a_{i}}{\underset{j=1}{\sum }}\left(x_{i}^{\left(j\right)}-\bar{x_{i}}\right){\left(x_{i}^{\left(j\right)}-\bar{x_{i}}\right)}^{\Phi }$$

where n is the total number of classes in the dataset and $x_{i}^{\left(a_{i}\right)}$ is the j th training set in the i th class. Based on this setting, we can obtain the inter-class scattering matrix:


$$\Pi_{\mathrm{IS}}=\overset{n}{\underset{i=1}{\sum }}a_{i}\left(K_{i}-\bar{K}\right){\left(K_{i}-\bar{K}\right)}^{\Phi }$$

where $a_{i}$ is the number of datasets of class i th, $K_{i}$ is the mean value of datasets of class i th, and $\bar{K}$ is the mean value of datasets, we can get the $\xi \left(C_{k}\right)$ value of the feature vector.


$$\xi \left(C_{k}\right)=\frac{\left|C_{k}^{T}\Pi_{\mathrm{IS}}C_{k}\right|}{C_{k}^{T}\Pi_{ICS}C_{k}}$$

where $C_{k}$ is the feature vector of the k th training set. We perform a decreasing rule on the $\xi \left(C_{k}\right)$ values to effectively order the feature vectors $C_{1}$ , $C_{2}$ , $C_{3}$ … $C_{a}$ to obtain a new set of feature vectors ϑ .


$$\vartheta =\left[\vartheta_{1},\vartheta_{2},\vartheta_{3}\cdots \vartheta_{a}\right]$$

where the $\vartheta_{1}$ eigenvector corresponds to the maximum value of $\xi \left(C_{k}\right)$ . By projecting the k th training set $x_{k}$ and test set $z_{k}$ onto the feature vector ϑ , the following can be obtained:


$$\theta_{k}=\vartheta^{T}x_{k}$$

$$\omega_{k}=\vartheta^{T}z_{k}$$

where $\theta_{k}$ is the projection of the training set counts on the feature vector ϑ and $\omega_{k}$ is the projection of the test set count on the feature vector ϑ . By employing the decreasing-rule principal component analysis (DR-PCA), we could derive a new set of eigenvectors with higher explanatory power. This enhancement facilitated a more accurate breakdown of the changes in mean HbO levels during fasting and glucose states in fNIRS data. Additionally, we implemented an advanced time series feature selection technique to optimize the model’s performance further. This involved employing a learning algorithm for feature search, which refined the selection of features based on the retention of the most optimal ones. Through this dual feature selection strategy, we could accurately identify the crucial components of the brain’s activity state, as depicted in Figure 5.

**Figure 5:**
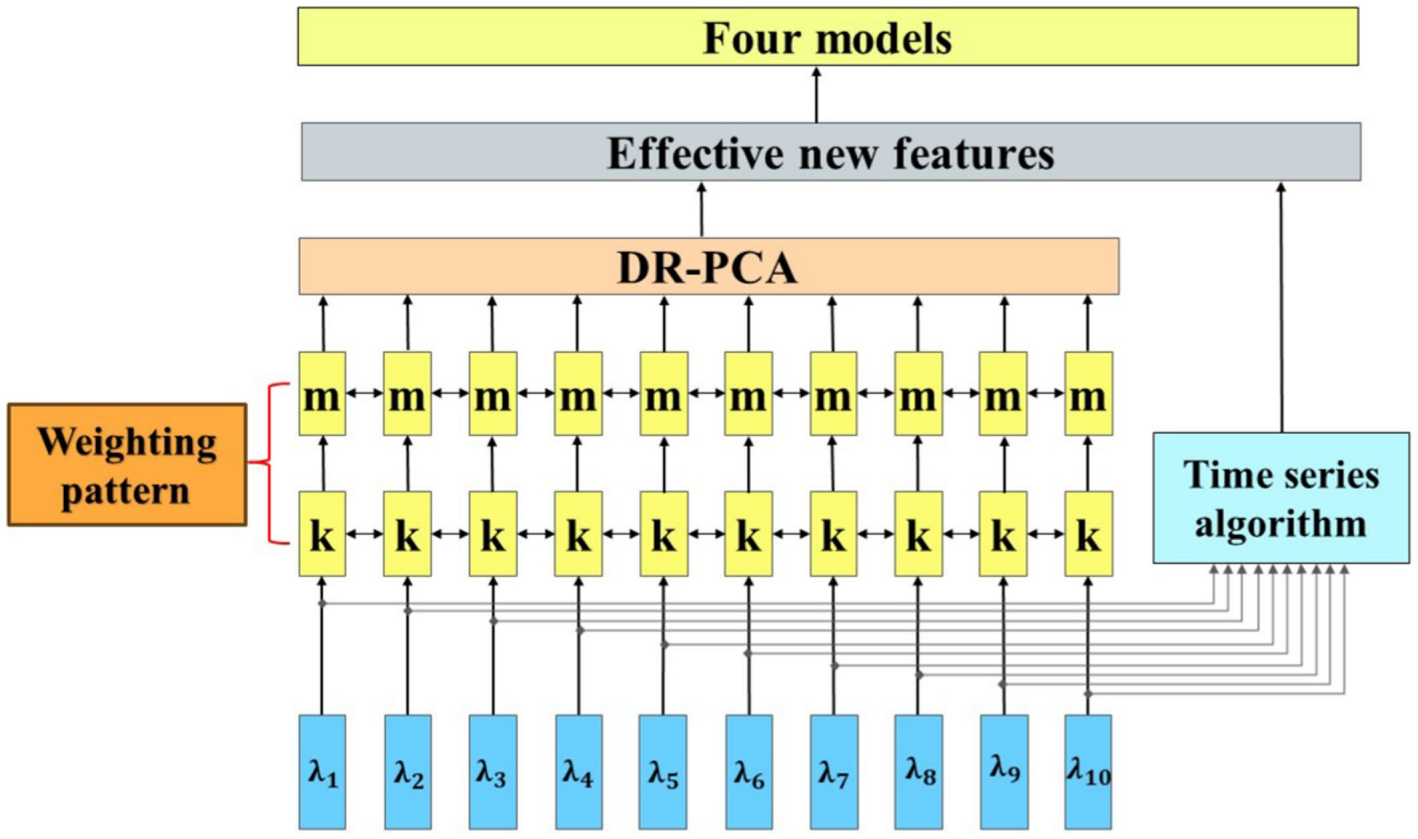
Flow chart of feature extraction.

**Figure 6:**
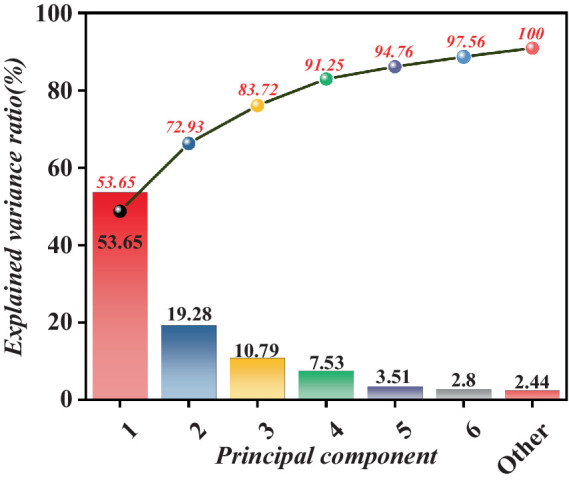
Characteristic contribution graph.

To optimize the learning algorithm even further and control its parameters effectively, a flexible weighting pattern was employed to adjust the degree of influence of different features on the model, whose feature contributions are shown in Figure 6. With this weighting scheme, it is possible to regulate the flexibility and robustness of the model more precisely, thus making its performance optimal.

As depicted in Figure 6, once the cumulative contribution rate surpasses a significant threshold of 90%, it is reasonable to infer that these principal components have effectively preserved the essential information, rendering the contribution of the remaining features marginal and inconsequential. This approach facilitates data reduction and extraction of crucial parts, thereby furnishing efficient and precise foundational elements for subsequent data analysis and model development.

### 2.5 fNIRS data and blood glucose correlation analysis

In this study, a Pearson correlation analysis (Hasan et al., 2020) was performed on the relationship between 10 features (*λ*_1_ to *λ*_10_) of fNIRS data and proper blood glucose levels in 15 subjects in fasting and glucose states. The aim was to investigate the potential relationship between fNIRS and blood glucose levels during glucose monitoring.

Assume that the fNIRS data is matrix X , dimension n×m , with each row representing a participant and each column representing a feature, n for samples and m for fNIRS data features. The blood glucose data matrix is G and dimension n×1 . from this, we calculate the mean vector as


$$\bar{X}=\frac{1}{n}\overset{n}{\underset{i=1}{\sum }}X_{i}$$

$$\bar{G}=\frac{1}{n}\overset{n}{\underset{i=1}{\sum }}G_{i}$$

where $X_{i}$ and $G_{i}$ denote the fNIRS data and blood glucose value of the *i*th sample, respectively. Then we obtained the centered data matrix


$$X_{c}=X-1{\bar{X}}^{T}$$

$$G_{c}=G-1{\bar{G}}^{T}$$

where 1 is an all-1 vector of size n×1 . We then compute the covariance matrix


$$S_{xy}=\frac{1}{n-1}{X_{c}}^{T}{G_{c}}^{T}$$

Next, we can obtain the self-covariance


$$S_{xx}=\frac{1}{n-1}{X_{c}}^{T}X_{c}$$

$$S_{yy}=\frac{1}{n-1}{G_{c}}^{T}G_{c}$$

We calculated the Pearson correlation coefficients of the 10 features (*λ*_1_ to *λ*_10_) of the fNIRS data with blood glucose values as


$$R=\frac{S_{xy}}{\sqrt{S_{xx}S_{yy}}}$$

By analyzing the correlation coefficient R , we can understand the degree of association between fNIRS data and blood glucose levels. In addition, to facilitate the comparison between fNIRS data and blood glucose levels, we normalized data *x*(*n*) using z-score zero-mean normalization to reduce individual differences (Xu et al., 2022).


$$y\left(n\right)=\frac{x\left(n\right)-\bar{x}}{\sigma }$$

where $\bar{x}$ is the mean of this data, σ is the standard deviation of this data, and the processed data $y\left(n\right)$ conforms to a standard normal distribution, resulting in the results of the correlation test between the fNIRS data and the blood glucose levels.

### 2.6 Predictive modeling

This study thoroughly explored a series of machine-learning techniques through optimal strategies to establish a reliable model for predicting blood glucose levels. These techniques include Random Forest (RF), Support Vector Machine (SVM), K-Nearest Neighbors (KNN), and XGBoost (Khan et al., 2021; Aouedi et al., 2022; Maher et al., 2023). We conducted a comprehensive evaluation of the effectiveness of each method in the context of this study. Through systematic exploration and comparison, we ultimately identified a meticulously tailored and effective model capable of reliably predicting blood glucose levels.

The training process for all models was carried out using nested leave-one-subject-out (LOSO) cross-validation, which incorporates both hyperparameter tuning and model evaluation (Blume et al., 2021; Wainer and Cawley, 2021). We divided the data collected from 15 participants into 15 datasets, each containing mean HbO concentration variations from a single participant. During each training iteration, the data from one participant (i.e., 1 fold) was retained as the test set, while the data from the remaining individuals (i.e., 14 folds) were used for model training. This process was repeated 15 times, with different participants tested each iteration. Additionally, we extensively exploit predictive error values to compute the mean, variance, and statistical distributions, thereby comprehensively understanding the model’s performance. We evaluate the model’s adaptability from both fitting error and prediction error perspectives, enabling a comprehensive assessment of the model’s performance across various aspects.

## 3 Results and analysis

### 3.1 Correlation of fNIRS data from the prefrontal cortex with blood glucose levels

**Figure 7:**
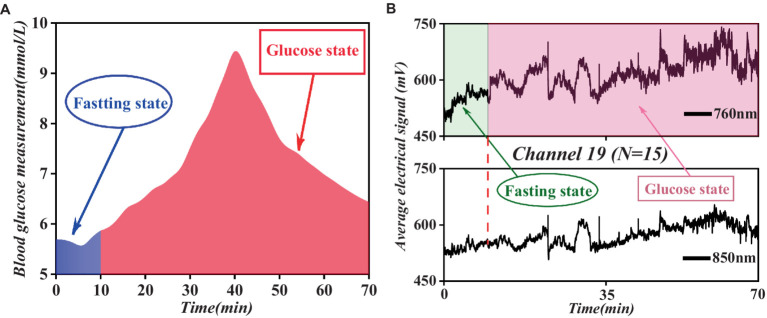
Comparative analysis of fasting and glucose states blood glucose levels and fNIRS spectral data trends. **(A)** Fasting and glucose states blood glucose levels. **(B)** Fasting and glucose states fNIRS spectral data in channel 19.

This study involved a comparison between the continuously monitored fNIRS mean data (from the 19th channel, which showed significant relevance) of all participants throughout the process and the mean blood glucose data of the control group, as illustrated in Figure 7.

As shown in Figure 7, the mean blood glucose level and fNIRS mean data maintained relatively low levels during the fasting state and displayed a stable trend. However, upon transitioning to the glucose state, both parameters experienced a rapid increase, reaching a peak within a specific timeframe, followed by a subsequent decrease to a certain range before stabilizing. This observed pattern underscores a correlation between blood glucose levels and fNIRS data.

To precisely quantify the correlation between fNIRS data and blood glucose levels, we performed Pearson correlation analysis, with the results presented in Table 3.

**Table 3:** Pearson correlation analysis.

	*G*
*λ*_1_	*r* = 0.401 *p* = 0.015
*λ*_2_	*r* = 0.193, *p* = 0.078
*λ*_3_	*r* = −0.063, *p* = 0.571
*λ*_4_	*r* = 0.005, *p* = 0.966
*λ*_5_	*r* = −0.150, *p* = 0.173
*λ*_6_	*r* = 0.128, *p* = 0.245
*λ*_7_	*r* = 0.324, *p* = 0.003
*λ*_8_	*r* = 0.463, *p* = 0.000
*λ*_9_	*r* = 0.572, *p* = 0.000
*λ*_10_	*r* = 0.692, *p* = 0.000

When analyzing the Pearson correlation results between the near-infrared features (*λ*_1_ to *λ*_10_) and blood glucose levels (*G*), we found that *λ*_8_, *λ*_9_, and *λ*_10_ displayed strong positive correlations (with respective r values of 0.463, 0.572, and 0.692) and highly significant statistical significance (all *p*-values were 0.000), indicating that they may be reliable indicators for monitoring or predicting changes in blood glucose. *λ*_1_ and *λ*_7_ also exhibited moderate positive correlations (with *r* values of 0.401 and 0.324, respectively) and statistical significance (with *p*-values of 0.015 and 0.003, respectively), while the correlations of other features (*λ*_2_, *λ*_3_, *λ*_4_, *λ*_5_, and *λ*_6_) were weaker and not statistically significant, implying that their association with blood glucose levels may not be as apparent or may require further research to determine their relevance. Based on experimental research involving continuous monitoring of blood glucose levels in the PFC using near-infrared spectroscopic imaging, we have preliminarily confirmed the feasibility of this technique in the field of blood glucose monitoring.

### 3.2 Mechanisms correlating changes in prefrontal hemoglobin concentration and blood glucose levels

**Figure 8:**
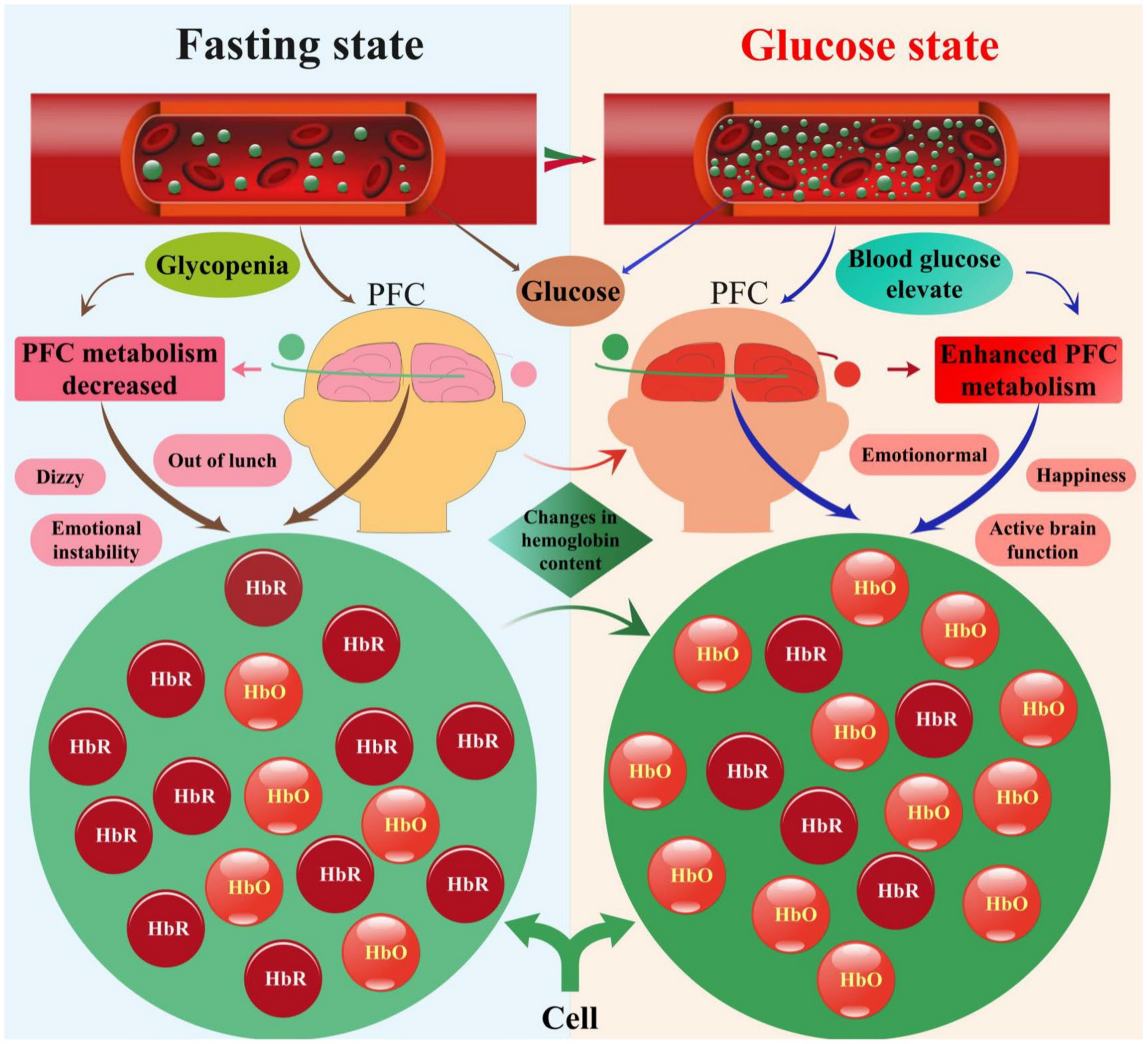
Visualization of PFC blood glucose-HbO-HbR correlation in the fasting and glucose states.

Our investigation focused on elucidating the mechanisms underlying the relationship between changes in prefrontal hemoglobin concentration and blood glucose levels, as depicted in Figure 8. Our analysis revealed dynamic fluctuations in prefrontal hemoglobin concentration in response to variations in blood glucose levels. Specifically, participants exhibited relatively stable prefrontal hemoglobin levels during the fasting state, indicating a baseline metabolic state. However, upon transitioning to the glucose state, a rapid increase in prefrontal hemoglobin concentration was observed, reaching peak levels within a specific timeframe. This surge in prefrontal hemoglobin levels suggests heightened metabolic activity in the PFC following glucose intake (Bini et al., 2022; Schwartz et al., 2023). Furthermore, this aligns with the results of the previous correlation analysis, indicating a direct relationship between cerebral metabolic activity and systemic glucose availability. Although we have proposed several possible mechanisms at this stage, further experimental research is needed to delve deeper into and validate the specific mechanisms. These findings provide new insights into understanding the effects of blood glucose changes on brain function and also offer a theoretical basis for utilizing fNIRS technology for glucose monitoring and management.

### 3.3 Impact of feature vectors of PCA and DR-PCA on the accuracy of prediction results

We conducted a series of comparison experiments to evaluate our proposed algorithm’s advancement and performance advantages in depth. First, we used the traditional PCA and DR-PCA algorithms as a control group and extracted 1 to 6 feature vectors, respectively (Ho et al., 2019). Subsequently, the extracted feature vectors were input into the KNN model for further processing, and finally, the respective forecast accuracies were obtained, as shown in Table 4.

**Table 4:** Forecast accuracy of PCA and DR-PCA feature vectors.

Number of feature vectors	PCA (accuracy/%)	DR-PCA (accuracy/%)
1	35.76	53.65
2	50.61	72.93
3	63.54	83.72
4	82.99	91.25
5	76.75	94.76
6	83.69	97.56

As indicated in Table 4, our proposed DR-PCA algorithm performs well with a different number of features. Compared to the traditional PCA method, the optimized algorithm exhibits superior performance and stability in feature extraction, leading to enhanced accuracy in subsequent forecast glucose levels. Furthermore, by incorporating the KNN model for further processing, we effectively integrate the critical aspects of feature extraction and forecast, improving algorithm performance. Specifically, we observe a progressive enhancement in forecast accuracy with the increase in the number of features, affirming the reliability and efficacy of our algorithm. Additionally, upon thorough analysis and application of the newly generated features, significant distinctions were observed in discerning brain activities in the fasting and glucose states, underscoring the effectiveness and precision of the feature selection method.

### 3.4 Prediction model accuracy and reliability analysis

**Figure 9:**
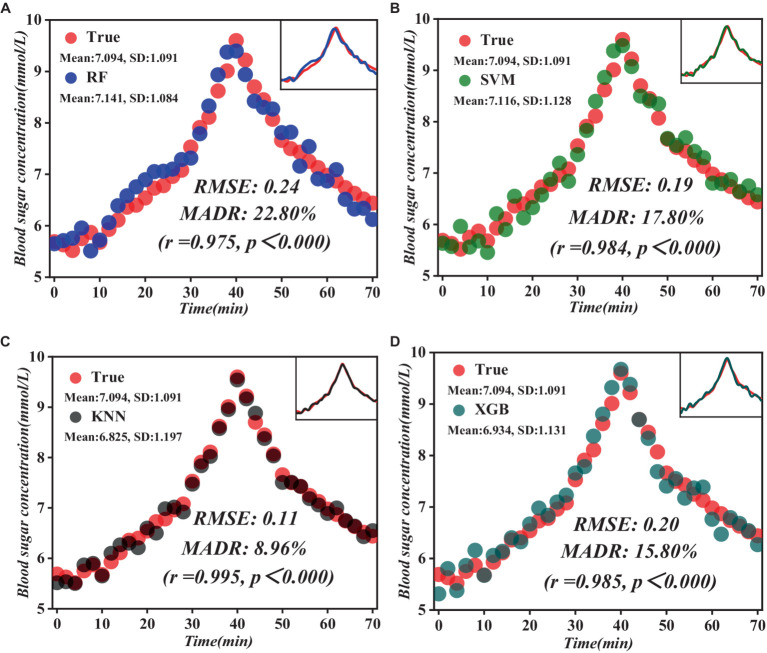
Comparison of the results of the prediction model with the measured blood glucose values. **(A)** RF prediction model results. **(B)** SVM prediction model results. **(C)** KNN prediction model results. **(D)** XGB prediction model results.

A series of validation trials were executed to validate the accuracy and stability of the constructed model fully. In these experiments, PFC spectral data was collected from the participants in real-time, and these data were inputted into the prediction model for real-time prediction of blood glucose levels. Simultaneously, the actual blood glucose values of the participants were calculated using the fingertip blood collection method to compare and analyze the prediction results with the exact values shown in Figure 9.

As evident from Figure 9, the KNN model shows significant advantages. Compared to the other three models, the KNN model showed lower root mean square error (RMSE) and mean absolute relative deviation (MARD) of 0.11 and 8.96%, respectively, and a highly significant positive correlation with actual blood glucose levels (*r* = 0.995, *p* < 0.000). By introducing advanced data processing techniques and model construction methods, we successfully broke through the limitations of traditional methods and realized more accurate and reliable prediction results. Through comparative experiments and practical validation, we verified that the prediction model we constructed is theoretically superior and achieves remarkable results in practical application, providing strong technical support for the accurate prediction of blood glucose levels.

**Figure 10:**
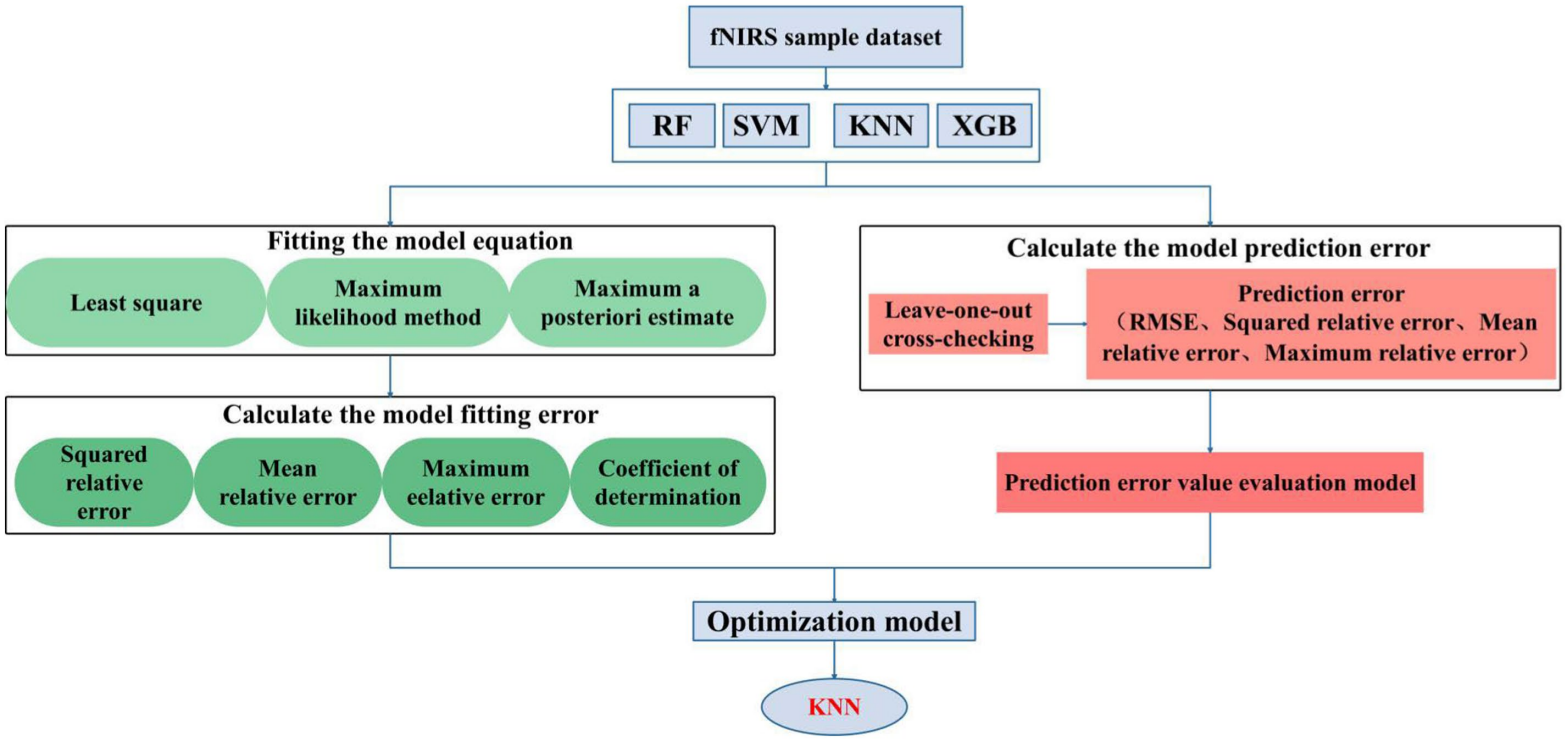
Flowchart of leave-one-out cross-validation method.

To gain deeper insights into the performance of the selected model, we employ a comprehensive approach for its evaluation, the results of which are illustrated in Figure 10.

As depicted in Figure 10, our approach is grounded in data-driven methodologies, employing a mathematical model to distill critical features from the dataset. The cross-validation method consistently refines the structure and parameters of the KNN model, ultimately designating it as the optimal choice. Furthermore, the model boasts non-invasiveness, precision, stability, continuous monitoring, and real-time alerts, ushering in new prospects for blood glucose monitoring and managing diabetic patients. Simultaneously, it provides a valuable reference for crafting personalized treatment regimens.

## 4 Results and discussion

Our study presents a groundbreaking approach to monitoring blood glucose levels using a portable, wearable fNIRS system. The correlation analysis between fNIRS data from PFC and blood glucose levels revealed significant insights. During fasting states, the average blood glucose levels and fNIRS data exhibited a stable trend, rapidly increasing upon transition to glucose states, peaking within a specific timeframe before stabilizing. This pattern underscores the potential of fNIRS technology in tracking glucose fluctuations, offering a non-invasive alternative to traditional blood glucose monitoring methods.

Furthermore, it is noteworthy that the strong correlation between *λ*_8_, *λ*_9_, and *λ*_10_ with blood glucose levels suggests that they may serve as reliable biomarkers for monitoring or predicting changes in blood glucose. Additionally, *λ*_1_ and *λ*_7_ also exhibit a certain degree of positive correlation and significance, indicating they may also hold research value. Conversely, other features (*λ*_2_–*λ*_6_) demonstrate weaker or non-significant correlations, suggesting their relationship with blood glucose levels may not be sufficiently close or may require further investigation with larger sample sizes. These findings are consistent with previous research, indicating the sensitivity of specific fNIRS data to changes in blood glucose concentration. However, our study extends these findings and confirms the feasibility of real-time monitoring using wearable fNIRS systems, representing a significant advancement compared to previous fixed-system approaches.

Our study delves into the complex interplay between PFC hemoglobin and blood glucose levels. It highlights how hypoglycemia affects PFC functionality and elucidates the body’s metabolic adjustments to maintain normal function. Through glucose intake, blood glucose levels rise, triggering increased metabolic activity in the PFC. This leads to changes in HbO and HbR concentrations, reflecting the PFC’s physiological adaptations to elevated blood glucose levels, which are essential for preserving brain function and modifying optical properties.

The design of our portable wearable fNIRS system represents a significant leap forward in the field of non-invasive glucose monitoring. Weighing ≤300 g and integrating a rechargeable lithium battery, the system offers true portability and comfort for indoor and outdoor experiments. This design overcomes the limitations of traditional, cumbersome fNIRS systems and ensures high-quality data acquisition by maintaining consistent contact with the scalp without restricting participants’ movements. In addition, we conducted a thorough comparative analysis of the critical technical parameters between existing fNIRS systems and our innovative engineered system, highlighting the latter’s significant advantages across various critical dimensions (see Table 5). Our system adopts the dual-wavelength near-infrared technology established in previous studies (Güven et al., 2020). We have expanded the number of channels, resulting in more intricate signal acquisition and higher resolution, thereby enhancing the sensitivity of biological signals. In our system, the higher fNIRS sampling rate adeptly captures the rapid dynamics of biological signals, offering unique benefits for real-time monitoring. Furthermore, optimizing photodetector spacing through multi-distance measurements enhances the accuracy of subcortical process monitoring. Our contemporary data transmission infrastructure ensures seamless real-time processing of extensive datasets, bolstering our capacity to conduct comprehensive, large-scale studies. The high-density measurement layout enables precise spatial mapping of biological signals and facilitates detailed examination of subtle changes between brain regions. Our system’s modular structure and intuitive interface significantly reduce operational complexity, allowing researchers to devote more attention to experimental design and data interpretation. Moreover, its compact, portable configuration is ideally suited for laboratory use and sets the stage for practical implementation in domains such as personalized healthcare and real-time emotion tracking. Our system exhibits significant performance enhancements through thorough comparative evaluation and innovative design and establishes a robust foundation for future research in brain-machine interfaces.

**Table 5:** Comparison of main parameters of fNIRS systems.

Parameters	Güven et al. (2020)	Gihyoun et al. (2023)	Al-Shargie et al. (2016)	Xu et al. (2023)	Buttafava et al. (2017)	This work
Wavelength (nm)	730, 850	780, 850	695, 830	762, 845.5	670, 830	760, 830
Channels	16	20	23	20	—	21
Sampling rate (Hz)	2	7.8	10	5	—	≤150
Photopolar spacing (mm)	25	—	30	—	30	10–55
Light source type	LED	LED	Laser	LED	Laser	LED
Detector type	Photodiode	—	APD	APD	SiPDs	SiPDs
Source-probe quantity	4, 10	16, 16	18, 16	—	2, 2	10, 8
Data transmission	Wireline transmission	Wireline transmission	Wireline transmission	Wireline transmission	Wireline transmission	Bluetooth wireless
High-density measurement	No support	Support	Support	Support	No support	Support
Operational complexity	Ordinary	Ordinary	Highly complex	Complex	Simple	Simple
Instrument power supply method	Direct plug-in or battery-powered with rechargeable batteries	Direct AC power supply	Direct AC power supply	Direct AC power supply	Direct AC power supply	Direct plug-in or battery-powered with rechargeable batteries
Portable and compact design	Wearable	Wearable	Wearable	Wearable	Wearable	Small, portable, and wearable

In addition, our study employed a series of comparative experiments to assess the performance of our proposed algorithm. The results indicate that the optimized algorithm demonstrates improved performance and stability in feature extraction, thereby enhancing the accuracy of subsequent blood glucose level predictions. We effectively enhanced the algorithm’s performance by integrating key feature extraction and prediction aspects via the KNN model. As the number of features increased, predictive accuracy gradually improved, affirming the reliability and effectiveness of our algorithm. Additionally, identifying brain activity differences between fasting and glucose states underscores the efficacy and precision of feature selection methods.

Our predictive modeling approach, utilizing RF, SVM, KNN, and XGBoost techniques, enabled us to construct a model for predicting blood glucose levels, and we evaluated the accuracy and stability of the model through nested leave-one-out cross-validation. The results indicate that the KNN model exhibited significant advantages in predicting blood glucose levels. This approach surpassed traditional predictive models by combining dual feature selection strategies, allowing for more precise identification of brain activity features associated with glucose levels. Specifically, our model demonstrated lower RMSE and MARD, at 0.11 and 8.96%, respectively. Compared to other non-invasive blood glucose monitoring systems, our system provides more accurate predictions that are closer to observed values (see Table 6). Furthermore, our system exhibited a highly significant positive correlation with actual blood glucose levels (*r* = 0.995, *p* < 0.000), further validating the reliability and effectiveness of our model.

**Table 6:** Comparison of main parameters of fNIRS systems.

Parameters	RMSE	MARD (%)	Correlation analysis
Xue et al. (2018)	0.114	—	*r* = 0.993, —
Meyhoefer et al. (2019)	—	—	*r* = 0.934, *p* < 0.000
Meyhöfer et al. (2020)	—	11.5	*r* = 0.934, *p* < 0.001
Tamada et al. (1999)	—	15.6	*r* = 0.880, —
Shokrekhodaei et al. (2021)	—	9.00	*r* = 0.980, —
Malchoff et al. (2002)	—	11.6	*r* = 0.870, —
This work	0.11	8.96	*r* = 0.995, *p* < 0.000

While our study provides innovative insights into the use of fNIRS technology in the field of blood glucose monitoring, it is essential to acknowledge its limitations. These include a relatively small sample size (only 15 participants), lack of diversity and representativeness in the sample, reliance on a single technical device, limited types of controlled variables, and absence of long-term monitoring data. These factors may affect the generalizability and depth of the results. Additionally, our research utilized a portable wearable fNIRS system for blood glucose monitoring. Although this system offers many advantages, its measurement accuracy and precision may not match traditional clinical standards. Future studies should focus on enhancing the system’s accuracy, exploring the use of advanced algorithms for data analysis, and conducting large-scale clinical trials to validate the effectiveness of this technology across different populations.

## 5 Conclusion

In this pioneering study, we have introduced and investigated a novel portable wearable fNIRS system for CGM, marking a significant advancement in the non-invasive tracking of glucose levels. Our research demonstrates the system’s efficacy in PFC activity with blood glucose levels, providing a promising new avenue for diabetes management and metabolic research. Through meticulous experimentation involving both fasting and glucose-loaded states among participants, we have established a robust correlation between PFC activity and blood glucose levels, as captured by our fNIRS system. This correlation, underscored by the KNN model that surpasses traditional CGM methods, highlights the potential of fNIRS technology in revolutionizing glucose monitoring by offering a non-invasive, real-time, and user-friendly alternative. Our findings underscore the feasibility of utilizing fNIRS for CGM and illuminate the intricate relationship between metabolic states and brain activity. Furthermore, developing a lightweight, portable, and highly accurate fNIRS system addresses the critical need for more adaptable and less intrusive CGM solutions. By overcoming the limitations of current technologies—such as invasiveness, the need for frequent calibration, and limited mobility—our system paves the way for enhanced patient compliance and broader applicability in daily life scenarios.

However, despite the potential significant connection between PFC and blood glucose regulation, the exploration of PFC activity during the CGM process using fNIRS remains in the preliminary exploratory and conceptual validation stage. This implies that, so far, the research community has not fully utilized fNIRS technology to systematically study this neurophysiological phenomenon. In the existing literature, reports on the real-time monitoring of brain activity related to glucose using fNIRS are relatively scarce and mostly confined to theoretical speculation or initial experimental stages. Therefore, this study aims to conduct a series of experiments to systematically apply fNIRS technology for the first time to explore PFC activity associated with changes in blood glucose levels, attempting to reveal how cognitive function interacts with glucose regulation. Through this pilot study with a limited sample size, we have preliminarily validated the feasibility of fNIRS application in this field and laid the groundwork for subsequent larger-scale and more in-depth research. Our goal is to evaluate whether fNIRS measurements of brain activity indices can reflect changes in blood glucose levels and explore its potential as a non-invasive CGM tool. Through this conceptual validation stage of research, we hope to provide scientific evidence and innovative insights for achieving more efficient and personalized blood glucose monitoring for future diabetic patients.

## Data availability statement

The datasets presented in this article are not readily available because they are subject to ongoing research. These datasets are provided with the permission of Shanghai University of Science and Technology and can be obtained by contacting the corresponding author. Additional data from this study are available in the article.

## Ethics statement

The studies involving humans were approved by Medical Ethics Committee, Peking University Sixth Hospital and University of Shanghai for Science and Technology Affiliated Shidong Hospital. The studies were conducted in accordance with the local legislation and institutional requirements. The participants provided their written informed consent to participate in this study.

## Author contributions

JC: Conceptualization, Formal analysis, Investigation, Methodology, Validation, Writing – original draft, Writing – review & editing. KY: Formal analysis, Software, Writing – review & editing. SZ: Funding acquisition, Project administration, Writing – review & editing. DZ: Funding acquisition, Resources, Supervision, Writing – review & editing.
